# DNA-Programmable Oligonucleotide Insecticide Eriola-11 Targets Mitochondrial 16S rRNA and Exhibits Strong Insecticidal Activity Against Woolly Apple Aphid (*Eriosoma lanigerum*) Hausmann

**DOI:** 10.3390/ijms26157486

**Published:** 2025-08-02

**Authors:** Vol Oberemok, Kate Laikova, Oksana Andreeva, Anastasia Dmitrienko, Tatiana Rybareva, Jamin Ali, Nikita Gal’chinsky

**Affiliations:** 1 Department of General Biology and Genetics, Institute of Biochemical Technologies, Ecology and Pharmacy, V.I. Vernadsky Crimean Federal University, 295007 Simferopol, Russia; voloberemok@gmail.com (V.O.); botan_icus@mail.ru (K.L.); andreeva-oksana.94.3@mail.ru (O.A.); dn2003@bk.ru (A.D.); 2 Laboratory of Entomology and Phytopathology, Dendrology and Landscape Architecture, Nikita Botanical Gardens—National Scientific Centre of the Russian Academy of Sciences, 298648 Yalta, Russia; diza_alex_a@mail.ru; 3 College of Plant Protection, Jilin Agricultural University, Changchun 130118, China; j.alirana@yahoo.com

**Keywords:** contact unmodified antisense DNA biotechnology (CUADb), DNA-programmable plant protection, *Eriosoma lanigerum*, ‘genetic zipper’ method, mitochondrial 16S rRNA, pest control

## Abstract

The potent and selective ‘genetic zipper’ method for insect pest control consists of three essential components: an antisense DNA (the finder), its complementary mature rRNA or pre-rRNA of the pest (the target), and the host’s endogenous DNA-guided rRNase (the degrader). Although this approach has been validated, the spectrum of effective rRNA targets remains insufficiently explored. In this study, we report for the first time the insecticidal efficacy of a novel oligonucleotide insecticide, Eriola-11, which targets the mitochondrial 16S rRNA of the woolly apple aphid *Eriosoma lanigerum* Hausmann. We hypothesized that the antisense-mediated silencing of mitochondrial rRNA would impair aphid viability and lead to physiological disruptions associated with mitochondrial energy metabolism. Eriola-11 was applied either once or twice (with a 24 h interval) to aphid-infested plants, and aphid mortality was recorded over 14 days. Mitochondrial 16S rRNA expression levels were quantified using molecular assays, and the degradation kinetics of Eriola-11 were assessed in aphid tissue homogenates. Results showed significant insecticidal activity, with 67.55% mortality after a single treatment and 83.35% after two treatments. Treated aphids exhibited the loss of their characteristic white woolly wax covering, and mitochondrial 16S rRNA expression was reduced 0.66-fold relative to the control. Additionally, Eriola-11 was fully degraded by aphid DNases from tissue homogenates within 3 h, highlighting its rapid biodegradability. These findings establish mitochondrial 16S rRNA as a viable target for antisense insecticides and expand the catalogue of potential rRNA-based targets, offering a promising avenue for environmentally sustainable pest control strategies.

## 1. Introduction

The woolly apple aphid *Eriosoma lanigerum* Hausmann (Hemiptera: Aphididae) is a well-studied and economically important pest of apple trees, particularly in young orchards. This phloem-feeding insect induces the formation of hypertrophic galls on both the aerial and underground plant parts, leading to structural deformities and physiological stress [1]. Colonies that establish on roots are protected from pesticide applications and environmental fluctuations, thereby serving as reservoirs for above-ground reinfestations in subsequent growing seasons [2,3,4]. Aphid-induced galls impede sap flow and frequently rupture, creating nutrient-rich feeding sites and vulnerable entry points for fungal pathogens, especially since *E. lanigerum* preferentially settles on damaged tissues [1]. Severe infestations, particularly under organic production systems, can significantly reduce photosynthesis, impair plant health, hinder bud formation, and increase susceptibility to disease [5]. Moreover, the accumulation of honeydew encourages the growth of sooty mould, and the white woolly wax produced by the aphid also contaminates the fruit. Together, these factors reduce marketability and cause overall yield losses [4,6,7,8,9,10,11,12,13,14]. As a result, *E. lanigerum* poses a major threat to sustainable apple production [15]. Therefore, efficient aphid control strategies are essential to minimize economic losses and ensure stable crop yields [16,17,18].

Integrated pest management (IPM) strategies in apple orchards increasingly emphasize biological control as an alternative to synthetic insecticides. The reduction in the use of broad-spectrum insecticides, such as carbamates and organophosphates, has facilitated the resurgence of natural enemies, particularly the parasitoid *Aphelinus mali*, a cornerstone species in biological control programmes targeting *E. lanigerum* [19,20]. Nevertheless, chemical insecticides remain the primary tool for pest management in many agricultural systems worldwide, including apple orchards [21,22,23]. However, excessive reliance on such chemicals has resulted in undesirable consequences, including pesticide resistance, non-target effects, and environmental pollution [24,25,26,27,28,29,30,31]. These challenges underscore the need for safer and more targeted pest control alternatives.

Among these emerging alternatives, oligonucleotide insecticides represent a promising and environmentally compatible solution. The ‘genetic zipper’ method based on short contact-unmodified antisense DNA biotechnology (CUADb) has gained attention for its high specificity and minimal off-target effects [32]. This approach utilizes antisense oligonucleotides designed to complement conserved regions of mature rRNA and/or pre-rRNA, thereby inhibiting protein biosynthesis. These oligonucleotide insecticides (referred to as “finders”) bind with high affinity and specificity to the target rRNA (the targets), recruiting DNA-guided rRNases (the degraders), like RNase H1, leading to the degradation of target rRNA in the RNA–DNA duplex. Given that ribosomal RNA (rRNA) constitutes approximately 80% of the total cellular RNA, in contrast to just ~5% for mRNAs [33], rRNA presents a high-yield target with a strong signal-to-noise ratio (~10^5^:1) and considerable variability, reducing the likelihood of off-target effects in non-target organisms [33,34,35]. Moreover, more than 60% of all energy is spent on the production and maintenance of ribosomes [36].

Importantly, research on Sternorrhynchan insects has demonstrated that oligonucleotide insecticides can induce both the upregulation and downregulation of target genes through a mechanism termed DNA containment (DNAc). This mechanism operates in two sequential steps: The first involves the arrest of target mature rRNA and/or pre-rRNA (formation of DNA–rRNA duplex), leading to a functional block of ribosomes and triggering the compensatory overexpression of rRNA via rDNA transcription. The second step of the DNA containment mechanism involves the degradation of the arrested mature rRNA and/or pre-rRNA via DNA-guided rRNase, like RNase H1 [37].

The CUADb strategy offers several important advantages. Oligonucleotide insecticides (briefly referred to as olinscides or DNA insecticides) degrade naturally in the environment, minimizing the risk of accumulation and resistance development. Current formulations of CUADb-based olinscides have demonstrated promising results, with mortality rates of 80–90% in various pest species within 3–14 days after a single treatment using a 100 ng/μL solution [38]. Previous studies have demonstrated the efficacy of CUADb-based insecticides targeting nuclear rRNAs, such as 28S and 18S rRNA, in multiple Sternorrhyncha pests, including *Unaspis euonymi*, *Dynaspidiotus britannicus*, *Icerya purchasi*, *Ceroplastes japonicus*, *Aonidia lauri*, *Coccus hesperidum*, and *Pseudococcus viburni* [38,39]. In addition, olinscides targeting internal transcribed spacer (ITS2) regions of pre-rRNA have shown effectiveness against *Macrosiphoniella sanborni*, *Schizolachnus pineti*, and *Trioza alacris* [32,34,40]. Even acaricidal effects have been observed against *Tetranychus urticae* [41]. Collectively, these findings suggest that CUADb may be applicable to 10–15% of all insect pest species with appropriate target selection and sequence optimization [34].

A promising new direction in this technology is the targeting of mitochondrial rRNAs—16S rRNA (~1140 nt) and 12S rRNA (~600 nt). Mitochondria are essential organelles responsible for adenosine triphosphate (ATP) synthesis via oxidative phosphorylation recruiting the ATP synthase complex by harnessing the proton gradient created by the electron transport chain, and are central to cellular bioenergetics [42]. While most mitochondrial proteins are encoded by nuclear DNA and synthesized in the cytoplasm, a few subunits of the ATP synthase complex are coded by mitochondrial DNA and synthesized by mitochondrial ribosomes, making rRNA a functionally very important target. Notably, our recent study on differential gene expression (DGE) analysis of *C. hesperidum* (4th day after contact application of oligonucleotide insecticide Coccus-11 targeting 28S rRNA) showed that during DNAc, most of investigated kinases are downregulated causing ‘kinase disaster’ in insect cells (including mTOR, a serine/threonine protein kinase playing crucial roles in the biogenesis of both cellular and mitochondrial ribosomes through the mTORC1 complex), while proteins from the mitochondrial ATP synthase complex and enzymes of the mitochondrial complex crucial for maintaining cellular energy (phosphoenolpyruvate carboxykinase (mitochondrial form), cytochrome c oxidase, Acyl-CoA dehydrogenase, NADH dehydrogenase (ubiquinone), succinate-CoA ligase, etc.) are upregulated, indicating the deficiency of cellular energy caused by oligonucleotide insecticide Coccus-11 [37,43]. Thus, mitochondrial rRNAs, 16S and 12S, are essential components of the mitochondrial ribosome and are present in high copy numbers, making them attractive and previously untapped targets for CUADb-based insecticides. Targeting mitochondrial rRNA can induce the systemic collapse of energy metabolism, thereby enhancing insecticidal efficacy.

In this study, we explore for the first time the insecticidal efficacy of a CUADb oligonucleotide—Eriola-11, designed to target the mitochondrial 16S rRNA of *E. lanigerum*. This work expands the utility of CUADb approach to include mitochondrial targets and evaluates the potential of Eriola-11 as a next-generation biotechnological tool for the sustainable management of this economically important pest.

## 2. Results

### 2.1. The Olinscide Eriola-11 Shows Pronounced Insecticidal Effects and Changes Morphology of E. lanigerum After Contact Application

**Figure 1:**
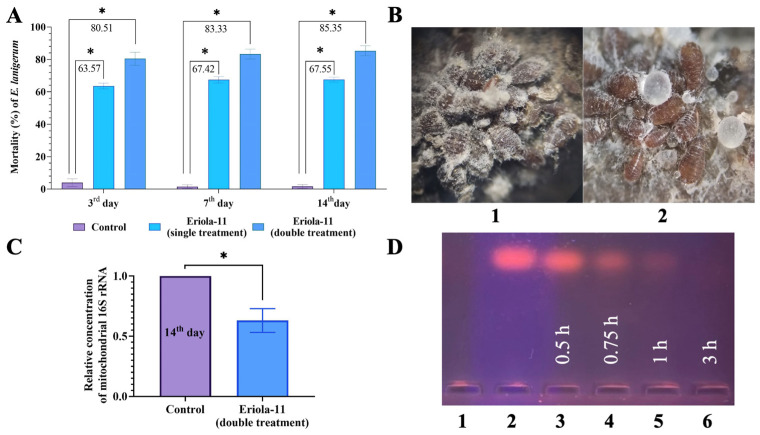
Experiments with oligonucleotide insecticide Eriola-11 on pest *E. lanigerum*. (**A**) Olinscide Eriola-11 shows pronounced insecticidal effect after contact application; Eriola-11—single treatment; Eriola-11—double treatment (with a 24 h interval); * is marked when *p* < 0.05. (**B**) Olinscide Eriola-11 changes morphology of *E. lanigerum*: *E. lanigerum* on an apple tree shoot (5× magnification) before (1) and after (2) treatment with Eriola-11; (**C**) Olinscide Eriola-11 significantly decreases the concentration of the mitochondrial 16S rRNA; expression of 16S rRNA was analyzed via RT-PCR; control was taken as 1 (100%); * is marked when *p* < 0.05. (**D**) Fast biodegradability of olinscide Eriola-11 via tissue homogenate deoxyribonucleases: 1—pure tissue homogenate of insects; 2—start (pure Eriola-11, 4 μg); 3—0.5 h; 4—0.75; 5—1 h; and 6—3 h.

The number of *E. lanigerum* individuals on apple shoots before a single treatment was 41.6 ± 2.8 individuals per 10 cm^2^ shoot (Figure 1). It was found that on the 3rd, 7th, and 14th days, the mortality of pest individuals after the use of Eriola-11 was 63.57 ± 1.79%, 67.42 ± 1.94%, and 67.55 ± 1.47%, respectively (*p* < 0.05) (Figure 1A). The characteristic white woolly wax of this species disappeared as a result of treatment with the olinscide Eriola-11, which is an interesting finding (Figure 1B).

The number of *E. lanigerum* individuals (larvae) before the double treatment with Eriola-11 averaged 132.3 ± 5.5 individuals per 10 cm^2^ shoot (Figure 1(B1)). After the treatment, dead individuals had a changed body colour (became dark grey and black, normally brown or red-brown) and completely lost their species-characteristic white woolly wax (Figure 1(B2)). The effectiveness of the first treatment averaged 66.18 ± 3.87% (*p* < 0.05). After the double treatment, viable individuals of *E. lanigerum* were found in areas of bark cracking, as well as under a layer of dead individuals. The effectiveness of the double treatment (with a 24 h interval) of olinscide Eriola-11 was 80.51 ± 4.03%, 83.33 ± 3.02%, and 85.35 ± 3.04% on the 3rd, 7th, and 14th days, respectively. In the water-treated control group, mortality comprised 11.5 ± 4.1%, 11.9 ± 3.9%, and 12.4 ± 3.9 on the 3rd, 7th, and 14th days, respectively (Figure 1A). Thus, double treatment with Eriola-11 (85.35 ± 3.04% mortality) was more efficient than single treatment by 18% (67.55 ± 1.47% mortality) at the end of the experiment (14th day).

### 2.2. Olinscide Eriola-11 Significantly Decreases the Concentration of the Mitochondrial 16S rRNA

Also, we evaluated the specificity of action of the olinscide Eriola-11 by studying the concentration of the target mitochondrial 16S rRNA. A decrease in the expression of the target gene is the gold standard for proof of the specificity of action for antisense oligonucleotides [44] and is also the characteristic feature of the second step of the DNA containment mechanism [39]. The expression of the mitochondrial 16S rRNA of *E. lanigerum* was analyzed on the 14th day after double treatment with olinscide Eriola-11. After treatment with the olinscide Eriola-11, the expression of the target mitochondrial 16S rRNA of the woolly apple aphid was significantly reduced 0.66-fold (*p* < 0.05) relative to the water-treated control group (Figure 1C).

### 2.3. Fast Biodegradability of Olinscide Eriola-11 via Tissue Homogenate Deoxyribonucleases

An analysis of the nuclease activity of woolly apple aphid tissue homogenates (Figure 1D) showed that within three hours, tissue DNases completely degraded the Eriola-11. The data obtained indicate the high biodegradability potential of such insecticides in ecosystems after their action on pests.

## 3. Discussion

This study aimed to explore the potential of mitochondrial 16S rRNA as a target for DNA-based insecticides. Our results strongly support the hypothesis that unmodified antisense oligonucleotides can effectively disrupt insect physiology by targeting mitochondrial rRNA. Specifically, we observed that the oligonucleotide insecticide Eriola-11, designed to target mitochondrial 16S rRNA in *E. lanigerum*, produced a significant insecticidal effect. By day 14, the mortality rate reached 67.55% after a single treatment and increased to 85.32% following a double treatment. These findings align with prior research demonstrating the efficacy of antisense oligonucleotides as insecticides against various pest species, including aphids [32,34,40]. The substantial mortality observed in this study underscores the potential of mitochondrial rRNA as a novel and effective target for pest control.

One of the key findings in this study was the enhanced efficacy of Eriola-11 when applied as a double treatment compared to a single application. The double treatment resulted in a higher mortality rate, which suggests that repeated exposure to the oligonucleotide insecticide enhances its insecticidal effect. This outcome is consistent with previous studies, such as Puzanova et al. [40], which also demonstrated that double treatments with oligonucleotide insecticide Macsan-11 improved the control of chrysanthemum aphid *M. sanborni*. The increased efficacy observed with double treatments may be attributed to the prolonged interaction of the oligonucleotide with the target rRNA, allowing for the greater disruption of essential mitochondrial processes. Therefore, our results suggest that treatment regimens using oligonucleotide insecticides like Eriola-11 should be optimized, potentially incorporating repeated applications to improve pest control outcomes.

Further supporting the effectiveness of Eriola-11 was the observed reduction in mitochondrial 16S rRNA expression in treated insects. Fourteen days post treatment, the target rRNA was reduced 0.66-fold compared to the water-treated control. This reduction indicates that the oligonucleotide successfully bound to its target rRNA and facilitated its degradation. This finding is consistent with the DNAc mechanism of action of antisense oligonucleotides in insects, which are known to bind to complementary RNA sequences and induce degradation through DNA-guided rRNase activity, including RNase H1 activity [37,39]. Notably, our recent DGE study on *C. hesperidum* after the application of oligonucleotide insecticide Coccus-11 (targeting 28S rRNA) showed that RNase H1 is also significantly upregulated during DNAc. RNase H1 functions independently of the cell cycle and cleaves RNA in RNA–DNA hybrids, including those formed between DNA and rRNA [37]. Thus, the observed reduction in 16S rRNA confirms the olinscide’s mode of action and its capacity to disrupt mitochondrial protein synthesis, which is essential for insect survival.

Another notable observation was the loss of the characteristic woolly white wax covering of *E. lanigerum* after treatment with Eriola-11. This change may be linked to the disruptions in mitochondrial biosynthesis, which is essential for the cellular production of energy. We assume that woolly white wax coverings serve as an energy storage substance [45], which may be utilized in response to the energy deficiency caused by mitochondrial dysfunction. The DGE study on *C. hesperidum* showed that during DNAc enzymes involved in the production of energy from lipids, namely triacylglycerol lipase (PNLIP), lysophospholipase III (LYPLA3), lysosomal acid lipase/cholesteryl ester hydrolase (LIPA), secretory phospholipase A2 (SPLA2), and phospholipase/carboxylesterase (IPR029058), were significantly upregulated. At the same time, crucial glycolytic enzymes were downregulated (pyruvate kinase, aldolase, and phosphofructokinase-1), while none of the glycolytic enzymes was upregulated, indicating a switch in energy synthesis from carbohydrates to lipids [43]. Additionally, some authors suggest that the primary role of secreted wax is to prevent the aphids from becoming contaminated by their own honeydew [46]. Therefore, wax-less aphids may become more vulnerable to honeydew exposure following Eriola-11 treatment. This loss of wax in our study suggests that the mitochondrial dysfunction caused by oligonucleotide targeting may interfere with the biosynthetic processes crucial for protective wax production.

The rapid biodegradation of Eriola-11 was another significant finding of this study. We observed that the oligonucleotide was completely degraded within three hours in the homogenate of *E. lanigerum* tissues. This degradation rate contrasts sharply with that of conventional chemical insecticides, which can persist in the environment for months or years [47]. The rapid biodegradation of Eriola-11 highlights its environmental safety, as it will be broken down by ubiquitous nucleases in ecosystems. This characteristic is particularly important for sustainable pest control strategies, where long-term environmental contamination by chemical pesticides remains a concern. The observed biodegradability of Eriola-11 is consistent with previous studies on oligonucleotide insecticides, which show faster degradation compared to synthetic chemicals [47]. This makes oligonucleotide insecticides a compelling alternative for environmentally responsible pest control.

In terms of practical application, the use of oligonucleotide insecticides such as Eriola-11 offers several advantages over traditional chemical insecticides. Firstly, oligonucleotide insecticides are highly specific, targeting only the pest species of interest without affecting non-target organisms, including beneficial insects [32]. This specificity minimizes ecological impact, making oligonucleotide insecticides a more environmentally friendly alternative. Additionally, the affordability of DNA insecticide production, facilitated by automatic DNA synthesizers, makes the large-scale production of these insecticides feasible [47]. The potential for the mass production of oligonucleotide insecticides opens up new possibilities for their widespread use in pest management, particularly in the context of IPM strategies [48].

Overall, our study demonstrates that mitochondrial 16S rRNA is a promising target for DNA-based insecticides, and that the oligonucleotide insecticide Eriola-11 shows substantial promise for pest control. Its high efficacy, environmental safety, and specificity highlight its potential as a viable alternative to conventional chemical insecticides. Future research should focus on refining treatment regimens, testing the effectiveness of oligonucleotide insecticides on a broader range of pests, and evaluating their integration into multi-faceted IPM strategies.

## 4. Materials and Methods

### 4.1. Insect

The aphid *E. lanigerum* (Hemiptera: Aphididae) was collected from apple trees (*Malus domestica* L.) in the Nikita Botanical Garden, Crimea (44°30′41.9″ N latitude and 34°13′57.3″ E) between July and October 2024. The degree of harmfulness of the *E. lanigerum* was determined by examining the bark, branches, petioles, bases of buds, and stems of the apple trees. Aphids were identified based on morphological characteristics; adult individuals (80%) and first instar nymphs (20%) were used directly in all experiments.

### 4.2. DNA Synthesis and Mass Spectrometry Analysis of Oligonucleotides

Oligonucleotides were synthesized using an automated DNA synthesizer ASM-800ET (BIOSSET, Novosibirsk, Russia) based on standard phosphoramidite chemistry on a universal solid support UnyLinker 500 Å (ChemGenes, Wilmington, MA, USA). Cleavage and deprotection were performed by incubating the oligonucleotides overnight at 55 °C in concentrated ammonia solution. The reaction mixture was subsequently filtered and evaporated using a vacuum rotary evaporator (Heidolph Instruments GmbH & Co. KG, Schwabach, Germany). The resulting residue was dissolved in deionized water (Merck Millipore, Molsheim, France) to the desired concentration, which was determined using the spectrophotometer NanoDrop Lite (Thermo Fisher Scientific, Waltham, MA, USA).

The molecular mass of the synthesized DNA sequences was determined via MALDI-TOF analysis mass spectrometry. Measurements were conducted in the positive-ion mode using 3-hydroxypicolinic acid as the matrix at a 2:1 matrix-to-analyte ratio on a LaserToF LT2 Plus mass spectrometer (Scientific Analysis Instruments, Manchester, UK). The theoretical *m*/*z* values were calculated using ChemDraw 18.0 software [49] (CambridgeSoft, Cambridge, MA, USA). All synthesized oligonucleotides were confirmed to match their expected structures. The measured *m*/*z* values differed from the theoretical values by no more than 10 units (Table 1).

### 4.3. Application of Eriola-11 as a Contact Insecticide

**Figure 2:**
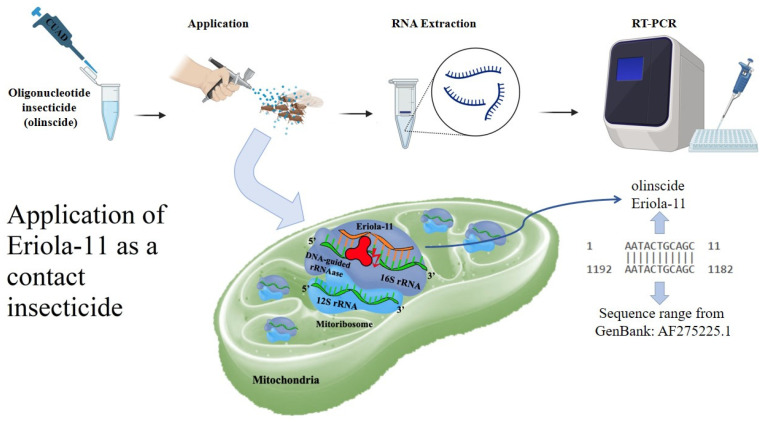
Schematic representation of the application and mode of action of Eriola-11 as a contact insecticide against *E. lanigerum*. Aphids were treated using a hand sprayer containing Eriola-11 (5′-AAT-ACT-GCA-GC-3′), an oligonucleotide-based insecticide designed to target mitochondrial 16S rRNA through a ‘genetic zipper’ mechanism, in which Eriola-11 serves as the finder strand, 16S rRNA as the target, and DNA-guided rRNase as the degrader. The solution was prepared in nuclease-free deionized water at a concentration of 100 ng/μL. A total of 1350 aphids, including first instar nymphs and adults, were treated across three independent replicates, with water-treated aphids serving as the control. Mortality was assessed on 3rd, 7th, and 14th day post treatment using a Micromed MS-4-ZOOM LED microscope. RNA was extracted from aphid samples, and RT-PCR analysis was conducted to verify the uptake and molecular activity of Eriola-11.

*E. lanigerum* was treated using a hand sprayer containing a solution of the oligonucleotide-based insecticide Eriola-11 (5′-AAT-ACT-GCA-GC-3′), which targets mitochondrial 16S rRNA (Figure 2). The solution was prepared in nuclease-free deionized water at a concentration of 100 ng/μL (1 mg per m^2^ of leaves with the pest). A water-treated group served as the control. A total of 1350 aphids (single and double treatment, 285 aphids and 1065 aphids, respectively), including first instar nymphs and adults, were treated across three independent experimental replicates. Aphid counts were recorded before treatment and on the 3rd, 7th, and 14th days post treatment. Mortality was assessed using a Micromed MS-4-ZOOM LED microscope with 0.75x-5x zoom lens (Micromed, Saint Petersburg, Russia).

### 4.4. Reverse Transcription and Real-Time PCR (RT-PCR) for 16S rRNA Quantification

Several studies have determined that ribosomal RNA represents the most stable reference genes, providing an accurate and reproducible real-time PCR assay [50,51,52]. On the 14th day, the total RNA was extracted using 50 *E. lanigerum* individuals from the experimental group and water-treated control group following treatment using the ExtractRNA reagent [53] (Evrogen, Moscow, Russia). Reverse transcription was performed using the MMLV Reverse Transcriptase kit [54] (Evrogen, Moscow, Russia) with the Eriola-R primer (200 ng per reaction). cDNA synthesis was carried out in a 20 μL reaction volume at 40 °C for 60 min, followed by enzyme inactivation at 70 °C for 10 min, using a LightCycler^®^96 Real-Time PCR System (Roche, Basel, Switzerland) (Table 2).

Real-time PCR was performed using SYBR Green I dye to amplify and quantify cDNA. The reaction mix included 3 μL of cDNA, 5X qPCRmix-HS SYBR master mix (Evrogen, Moscow, Russia), and gene-specific primers (Table 2). Amplification was conducted under the following thermal profile: initial denaturation at 95 °C for 10 min, followed by 45 cycles of denaturation at 95 °C for 10 s, annealing at 50 °C for 15 s, and elongation at 72 °C for 10 s [55]. All reactions were performed in triplicate. Melt curve analysis was used to confirm the specificity of amplification and absence of the non-specific products.

### 4.5. Analysis of DNase Activity

DNase activity in *E. lanigerum* tissue homogenates was analyzed by incubating 5 mg of insect tissue (ca. 5 insect individuals) in 10 μL of Milli-Q deionized water (Millipore, Molsheim, France), followed by the addition of 10 μL of Eriola-11 at a concentration of 400 ng/μL. The homogenate was incubated at 27 °C in a solid-state thermostat for 30, 45, 60, and 180 min. Subsequently, samples were heated at 90 °C for 60 min to denature protein and centrifuged at 12,000× *g* for 1 min. After that, 10 µL of the homogenate with Eriola-11 was mixed with 3 µL of 4X Gel Loading Dye, Blue (Evrogen, Moscow, Russia) and loaded onto a 1.8% agarose gel prepared with standard TBE buffer and stained with ethidium bromide (EB) (10–15 μL of EB at a concentration of 10 mg/mL per 55 mL of agarose gel). Electrophoresis was carried out at 10 V/cm for 40 min using a BlueMarine electrophoresis system (SERVA Electrophoresis GmbH, Heidelberg, Germany) and Mini-300 power supply (Major Science, Taoyuan City, Taiwan). DNA bands were visualized under a transilluminator (Vilber Lourmat, Marne-la-Vallée, France) [56].

### 4.6. Statistical Analyses

The mean and standard error of the mean (SE) were calculated using Student’s *t*-test. Control was taken as 1 (100%) for gene expression analysis. The graph shows the mean values and standard errors for the three replicates compared to the water-treated control group (Figure 1A,C). All above-mentioned calculations were performed using GraphPad Prism 9 software [57] (GraphPad Software Inc., Boston, MA, USA).

## 5. Conclusions

The CUADb-based ‘genetic zipper’ method offers a simple and efficient approach for pest control, requiring the synthesis of a complementary DNA strand (oligonucleotide insecticide) targeting mature rRNA and/or pre-rRNA, such as mitochondrial 16S rRNA. These insecticides can be applied topically, and the method is highly adaptable through tools like the DNAInsector web application (dnainsector.com). This study demonstrates that the Eriola-11 oligonucleotide insecticide effectively targets *E. lanigerum*, causing significant insect mortality and the loss of its protective waxy covering (possible compensation of decrease in ATP production via mitochondria through consumption of waxes as energy storage substances). The insecticide biodegrades completely within three hours in insect tissue homogenates, highlighting its rapid environmental degradation. The affordability of DNA insecticide production and their scalability through automatic DNA synthesizers and liquid phase synthesis make CUADb-based oligonucleotide insecticides promising for large-scale pest control. These DNA-based solutions offer a competitive alternative to traditional chemical insecticides, with the potential to address microevolution in pests. Moreover, the biodegradation of these insecticides is much faster than with conventional chemicals, posing a lower environmental risk. CUADb shows promise for creating a sustainable, xenobiotic-free future in agriculture, positioning oligonucleotide pesticides as prospective pest control agents.

## Figures and Tables

**Table 1:** Results of the analysis of synthesized oligonucleotides using the MALDI-TOF method.

Olinscides	Theoretical *m*/*z* Ratio	Received *m*/*z* Ratio
Eriola-11	3323.61	3329.56
**Primers**	**Theoretical *m*/*z* Ratio**	**Received *m*/*z* Ratio**
Eriola-F	5511.96	5505.47
Eriola-R	5373.92	5365.84

**Table 2:** Primer sequences used for real-time PCR amplification of *E. lanigerum* 16S rRNA.

Gene	Primer	Primer Sequence (5′-3′)	T_m_	PCR Product	GenBank (Sequence ID)
*16S rRNA*	Eriola-F	TATAGGATCTGCTCAATG	50 °C	100 bp	AF275225.1
	Eriola-R	TCTTTCATTCAAGTCTCC			

## Data Availability

The original contributions presented in this study are included in the article. Further inquiries can be directed to the corresponding authors.
